# Breast Milk Is a Potential Reservoir for Livestock-Associated *Staphylococcus aureus* and Community-Associated *Staphylococcus aureus* in Shanghai, China

**DOI:** 10.3389/fmicb.2017.02639

**Published:** 2018-01-11

**Authors:** Xiaoliang Li, Yun Zhou, Xianlin Zhan, Weichun Huang, Xing Wang

**Affiliations:** ^1^Department of Laboratory Medicine, Shanghai Children's Medical Center, Shanghai Jiaotong University School of Medicine, Shanghai, China; ^2^Department of Intensive Care Unit, Huashan Hospital, Fudan University, Shanghai, China; ^3^Department of Laboratory Medicine, The 455th Hospital of Chinese People's Liberation Army, Shanghai, China

**Keywords:** breast milk, livestock-associated *Staphylococcus aureus*, community-associated *Staphylococcus aureus*, prevalence, antibiotic resistance

## Abstract

Breast milk is the first choice in feeding newborn infants and provides multiple benefits for their growth and development. *Staphylococcus aureus* usually exists in breast milk and is considered one of the most important causative infective agents. To be effective in preventing and controlling *S. aureus* infections among infants, the aim of this study was to determine the occurrence and molecular characteristics of *S. aureus* isolated from 1102 samples of breast milk between 2015 and 2016 in Shanghai, China. Out of 71 *S. aureus* strains isolated, 15 (21.1%, 15/71) were MRSA and all the strains were characterized by *spa* typing, Multi-Locus Sequence Typing, SCC*mec* typing, antibiotic resistance testing and virulence-associated genes. A total of 18 distinct sequence types (STs) and 36 *spa* types were identified within the 71 isolates, among which the most frequently represented was ST398 (19.7%, 14/71), followed by ST7 (18.3%, 13/71), ST59 (16.9%, 12/71). The three predominant STs accounted for more than one half of all *S. aureus* isolates. The most prevalent *spa* types were *t*091 (12.7%, 9/71), followed by *t*571 (8.5%, 6/71), *t*189 (7.0%, 5/71), *t*034 (5.6%, 4/71), *t*437 (5.6%, 4/71), and *t*701 (4.2%, 3/71). All MRSA isolates belonged to SCC*mec* IV and V, accounting for 66.7 and 33.3% respectively. Notably, 23 (32.4%) *S. aureus* strains were multidrug resistance (MDR), including 4 (5.6%) MRSA and 19 (26.8%) MSSA strains, and MDR isolates were mostly resistant to penicillin, erythromycin and clindamycin. All isolates exhibited simultaneous carriage of at least 5 of 33 possible virulence genes and the most prevalent genes detected were *icaA* (100%), *clfA* (100%), *hla* (100%), *sdrC* (94.4%), *hlg*2 (88.7%), *lukE* (57.8%). 39 (54.9%, 39/71) isolates, including 9 (12.7%) of MRSA isolates, harbored ≥10 tested virulence genes evaluated in this study. The *pvl* gene was detected in 8 strains, which represented 5 different STs, with ST59 being the most one. Overall, our findings showed that *S. aureus* strains isolated from breast milk were mainly MSSA (78.9%, 56/71) and exhibited high genetic diversity in Shanghai area of China. Breast milk was a reservoir for LA-SA (ST398) and CA-SA (ST59), which was likely a vehicle for transmission of multidrug-resistant *S. aureus* and MRSA lineages. This is a potential public health risk and highlights the need for good hygiene practices to reduce the risk of infant infections.

## Introduction

Breast milk is recognized as the best food for newborn infants, which contains all the nutrients that are essential to the children in the first 6 months and favors the development of the immune system (Albesharat et al., 2011). However, breast milk is not sterile and represents a complex ecosystem with a considerable diversity of bacteria instead. It is well known to be colonized by benefical flora with a majority of bifidobacteria, promoting development of infant's healthy gut microbiota. Not surprisingly, it may contains potentially pathogenic bacteria species (Barbosa-Cesnik et al., 2003). In addition, the collection, storage and transport of breast milk may introduce pathogenic contamination, increasing the risk of infection to these vulnerable premature infants. In fact, breast milk has been reported to act as a repository of bacteria for vertical transmission from mother to infant. *Staphylococcus aureus* is the most frequently isolated pathogenic bacteria in breast milk (Barbosa-Cesnik et al., 2003) and could cause a wide variety of infections including pneumonia, sepsis, skin lesion and food poisoning among infants.

*S. aureus* is a common colonizer of skin and mucous membranes in human and animals, and 30–50% of healthy adults are colonized with it during their lifetime. *S aureus* infection occurs following breaks in skin or mucosal barriers, ranging from mild skin and soft-tissue infections to severe systemic infections such as sepsis and necrotizing pneumonia (Lowy, 1998). *S. aureus* has been recognized as a major cause of hospital-associated (HA) infections worldwide firstly, thereafter it transferred into the communities and became an important causative agent of community-associated (CA) infections (Mediavilla et al., 2012; Li et al., 2016). Recently, *S. aureus* has been identified as an emerging pathogen in livestock, companion animals and humans in contact with livestock, which called livestock-associated *S. aureus* (LA-SA) (Fitzgerald, 2012). Besides, *S. aureus* strains have been reported in animal-source food such as meat, fish, milk and dairy products (Wang et al., 2014), suggesting these foods may serve as reservoirs and sources of community-associated *S. aureus* (CA-SA). So far, it has become a particular public threat to human and animal health. There are some differences between HA-MRSA, CA-MRSA, and LA-MRSA in molecular characteristics (Chuang and Huang, 2013; Chen and Huang, 2014). HA-MRSA isolates typically harbor relatively large SCC*mec* elements (types I-III), and are resistant to multiple antibiotics, including β -lactams. CA-MRSA isolates usually carry smaller SCC*mec* elements (types IV-V) and are only resistant to β-lactam antimicrobials and possess different exotoxin gene profile. Most LA-MRSA strains are host-specific and contain variable mobile genetic element (MGEs). In China, ST239 and ST5 are predominant HA-MRSA clones (Xiao et al., 2013), ST59 is the most prevalent CA-MRSA clone (Qiao et al., 2013; Chen and Huang, 2014), while ST9, ST97, and ST398 are the common LA-MRSA clones (Cui et al., 2009; Wang et al., 2015). The spreading of epidemic clones among the hospital, the community and the livestock environment makes the distinction among CA-MRSA, HA-MRSA, and LA-MRSA become blurred.

It has been reported that breastfeeding was associated with severe neonatal disease, including infantile pneumonia, neonatal sepsis and food poisoning (Le Thomas et al., 2001; Kayiran et al., 2014). However, there has been no recommendation to examine breast milk routinely for pathogenic bacteria when a mother feeds her own baby. So far, fewer data are available regarding the prevalence of *S. aureus* and MRSA in breast milk. The aim of this study was to determine the prevalence, antibiotic resistance, and molecular characteristics of *S. aureus* and MRSA isolated from breast milk samples between 2015 and 2016 in Shanghai. Such information could provide guidance for further clinical and epidemiologic studies, rational usage of antimicrobial agents.

## Materials and methods

### Sample collection and bacterial isolation

From January 2015 to December 2016, a total of 1102 breast milk samples were collected from pediatric patients' mothers in a university hospital in Shanghai (Shanghai Children's Medical Center, affiliated with Shanghai Jiao Tong University). For milk collection, the breast of these mothers were cleaned with water and dried. Cotton swabs with 70% ethanol were used to disinfect the surfaces of the breast. The first few streams of milk were dropped. The collected milk was kept in a cooler with ice and transported to the laboratory within 2 h. The milk samples were cultured on 5% blood plate and inoculated at 37°C for 24 h. *S. aureus* isolates were confirmed by classic microbiological methods: Gram stain and catalase and coagulase activity on rabbit plasma. They were further identified by biochemical characterization using the Api-Staph test (bioMérieux, Lyon, France). All *S. aureus* isolates recovered from breast milk samples were each from a separate mother. These isolates were processed in Class II Biological Safety Cabinets. All strains were stored at −70°C and grown overnight on sheep blood agar plates at 37°C.

This study was approved by the Ethics Committee of Shanghai Children's Medical Center, and all isolates were collected with informed consents prior to sample collection.

### Antimicrobial susceptibility testing

The antibiotic susceptibility profiles of all *S. aureus* isolates in the current study were performed using the bioMe'rieux VITEK2 system following manufacturer's instructions. Results were interpreted according to the recommendations and definitions of the Clinical and Laboratory Standards Institute (CLSI, 2015). The following 17 drugs were tested: cefoxitin (FOX), linezolid (LZD), ciprofloxacin (CIP), clindamycin (DA), erythromycin (E), trimethoprim-sulfamethoxazole (SXT), moxifloxacin (MOF), nitrofurantoin (FD), vancomycin (V), tetracycline (TET), penicillin (P), rifampicin (RF), levofloxacin (LVX), ampicillin (AMP), gentamicin (GM), quinupristin/dalfopristin (Q/D), and tigecycline (TGC). *S. aureus* ATCC 29213 was used as a quality control.

### MLST analysis

All *S. aureus* isolates were performed according to the protocol of Enright (Enright and Spratt, 1999) on the *S. aureus* MLST website (http://saureus.mlst.net) to detect the following seven housekeeping genes (Aanensen and Spratt, 2005): carbamate kinase (*arcC*), shikimate dehydrogenase (*aroE*), glycerol kinase (*glp*), guanylate kinase (*gmk*), phosphate acetyltransferase (*pta*), triosephosphate isomerase (*tpi*), and acetyl coenzyme A acetyltransferase (*yqiL*). PCR amplicons of seven *S. aureus* housekeeping genes were obtained from chromosomal DNA. The sequences of the PCR products were compared with the existing alleles available from the MLST website, and alleles and ST were assigned by submitting the sequences. Clustering of related STs, which were defined as clonal complexes (CCs), was determined using eBURST (based on related STs).

### SCC*mec* typing

Staphylococcal cassette chromosome *mec* (SCC*mec*) typing was carried out discriminating the *mec* complex and the cassette chromosome recombinase(*ccr)* genes complex as described elsewhere (Kondo et al., 2007), which was based on a set of multiplex PCRs (M-PCRs) with 14 primers. SCC*mec* types I–V were assigned according to the combination of the *ccr* type and *mec* class. MRSA isolates that could not be assigned to any expected type were defined as nontypable (NT).

### *Spa* typing

In *S. aureus*, the polymorphic X region of staphylococcal protein A (*spa)* gene consists of a variable number of 24 bp repeat units (Shopsin et al., 1999) that allow isolates to be distinguished from one another. The *spa* typing was based on variations of the repeat units. Amplification and sequencing of the X region were performed as described previously by Koreen et al. (2004). The *spa* typing was assigned by submitting the data to the *S. aureus spa* type database (http://www.ridom.de/spa-server/).

### Detection of virulence genes

All *S. aureus* isolates were subjected to a multiplex PCR assay for the detection of 33 staphylococcal virulence genes: the staphylococcal enterotoxin genes (*sea, seb, sec, sed, see, seg, seh, sei, sej, sel, sem, sen, seo, sep, seq, sek*), the toxic shock syndrome toxin (*tsst*), the arginine catabolic mobile gene(*arcA*), the exfoliative toxin genes (*eta, etb*), the leukocidin (*luk*F/S-PV, *luk*E, *luk*M) (Lina et al., 1999), the bacteriocin (*bsaA*), the hemolysin gene (*hla, hlb, hlg, hlg2*), and the adhesin genes (*clfA, icaA, sdrC, sdrD*, and *sdrE*) as previously described (Arvidson and Tegmark, 2001; Peacock et al., 2002; Bubeck Wardenburg et al., 2007).

### Statistical analysis

Statistical analyses were performed using Stata software (version 10.1/SE, Stata Corp, College Station, TX, USA). We used the χ^2^ and Fisher's exact tests, as appropriate for analysis of categorical data. Statistical significance was set at *P* ≤ 0.05.

## Results

### Prevalence of *S. aureus* and MRSA in breast milk

Overall 1102 breast milk samples, collected from a university hospital in Shanghai between 2015 and 2016, were subjected to bacteriological analysis. Seventy-one (6.4%, 71/1102) strains of *S. aureus* isolated from 71 breast milk, 15 (21.1%, 15/71) were MRSA. PCR assay for *mecA* and disk diffusion test with oxacillin confirmed methicillin resistance of 15 isolates.

### MLST, SCC*mec*, and *spa* typing

The evolutionary and genetic diversity of *S. aureus* isolates within breast milk was analyzed by MLST (Table 1). There were 18 distinct STs identified within the 71 isolates, among which the most frequently represented was ST398 (19.7%, 14/71), followed by ST7 (18.3%, 13/71), ST59 (16.9%, 12/71). These three predominant STs accounted for more than one half of all *S. aureus* isolates. Other STs represented included ST188 (7.0%, 5/71) and ST6 (7.0%, 5/71) with five isolates, ST1 (4.2%, 3/71) and ST5 (4.2%, 3/71) with three isolates, ST15 (2.8%, 2/71), ST20 (2.8%, 2/71), ST88 (2.8%, 2/71), ST615 (2.8%, 2/71), and ST630 (2.8%, 2/71) with two isolates, and 6STs (ST8, ST12, ST22, ST25, ST508, and ST1290) with one isolate. Eight isolates harboring *pvl* were distributed among 5 different STs, including ST59 (4 isolates) as well as ST188, ST1, ST615, ST22 (1 isolate each).

**Table 1:** Clonal complexes and the relationship among the molecular types of 71 *S. aureus* isolates recovered from breast milk.

				**SCC*****mec*** **type**		
	**Clonal complex (CC)**	**MLST (*n*, %)**	***spa* Type**	**IV**	**V**	**NO**.
MSSA (56, 78.9%)	CC7	ST7 (13, 18.3%)	*t*091			9
			*t*796			2
			*t*1685			1
			*t*14204			1
	CC398	ST398 (9, 12.7%)	*t*034			2
			*t*571			4
			*t*2582			1
			*t*6606			1
			*t*7160			1
	CC59	ST59 (7, 9.9%)	*t*172			2
			*t*437			3
			*t*441			1
			*t*3736			1
	CC1	ST1 (2, 2.8%)	*t*127			1
			*t*286			1
		ST188 (4, 5.6%)	*t*189			4
		ST1290 (1, 1.4%)	*t*131			1
	CC5	ST5 (3, 4.2%)	*t*002			1
			*t*535			1
			*t*954			1
		ST6 (5, 7.0%)	*t*701			3
			*t*12306			1
			*t*14164			1
	CC72	ST8 (1, 1.4%)	*t*9101			1
		ST615 (1, 1.4%)	*t*148			1
		ST630 (2, 2.8%)	*t*3930			1
			*t*3386			1
	CC15	ST15 (2, 2.8%)	*t*084			1
			*t*085			1
	CC20	ST20 (2, 2.8%)	*t*164			2
	CC12	ST12 (1, 1.4%)	*t*4176			1
	CC22	ST22 (1, 1.4%)	*t*309			1
	CC25	ST25 (1, 1.4%)	*t*078			1
	CC45	ST508 (1, 1.4%)	*t*2334			1
MRSA (15, 21.1%)	CC398	ST398 (5, 7.0%)	*t*034		2	2
			*t*571	1	1	2
			*t*6606		1	1
	CC59	ST59 (5, 7.0%)	*t*172	3		3
			*t*437	1		1
			*t*3736	1		1
	CC1	ST1 (1, 1.4%)	*t*127	1		1
		ST188 (1, 1.4%)	*t*189	1		1
	CC72	ST615 (1, 1.4%)	*t*324	1		1
	CC78	ST88 (2, 2.8%)	*t*15319		1	1
			NT	1		1

Thirty-six *spa* types were observed among the 71 isolates. The most prevalent *spa* types were *t*091 (12.7%, 9/71), followed by *t*571 (8.5%, 6/71), *t*189 (7.0%, 5/71), *t*034 (5.6%, 4/71), *t*437 (5.6%, 4/71), *t*701 (4.2%, 3/71). Each of the remaining *spa* types was represented in less than three isolates.

**Figure 1:**
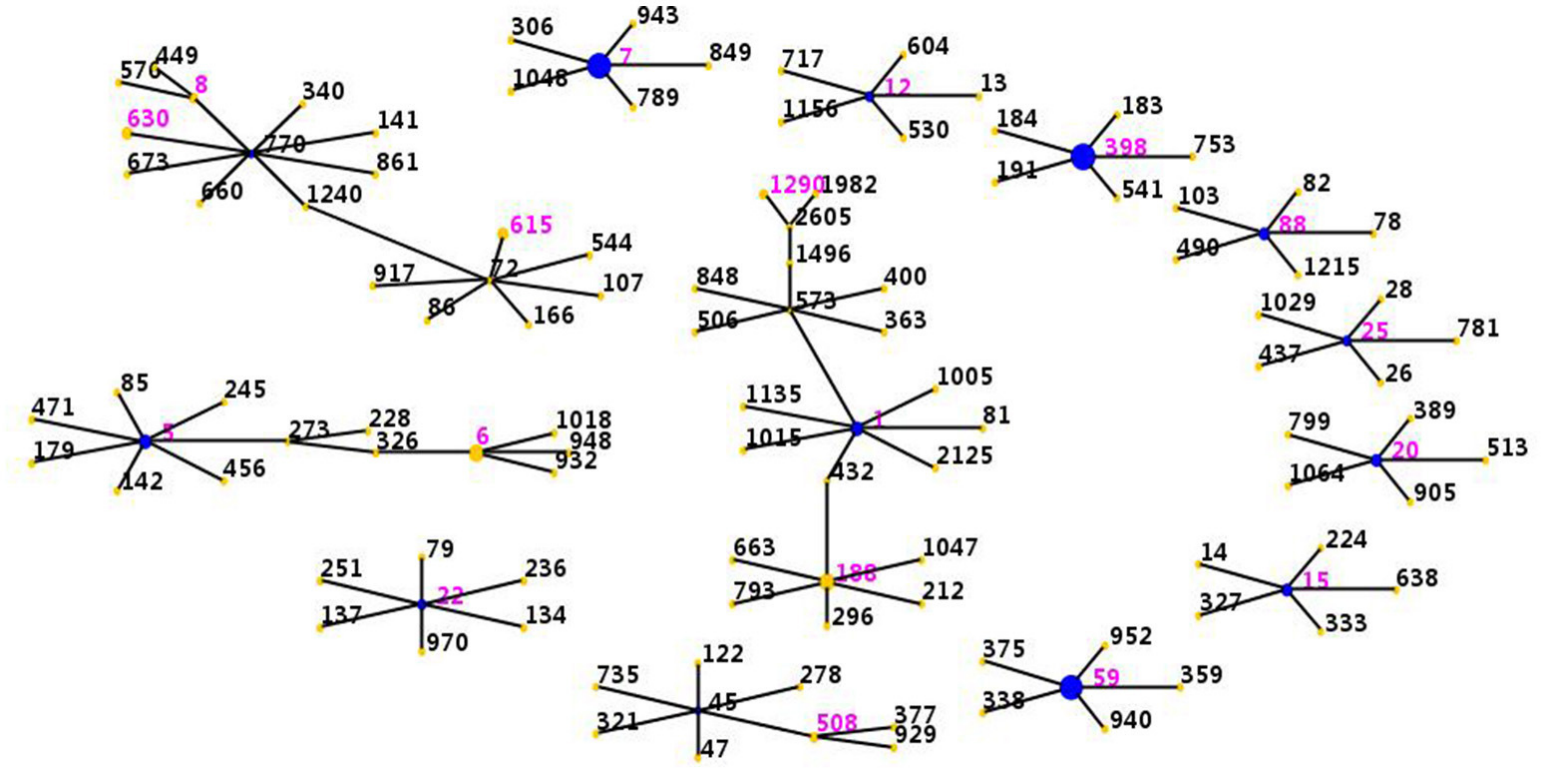
Distribution of STs in the clonal complexes. The eBURST application of the MLST data from all of the isolates analyzed in this study. The purple numbers represent 18 STs which are found in 71 *S. aureus* isolates recovered from breast milk between 2015 and 2016 in Shanghai, China. STs that are linked by a line belong to the same cluster. Circle sizes are proportional to the number of strains within the ST.

The eBURST analysis was performed on all the *S. aureus* isolates by using all STs available in the MLST database was shown. This methodology revealed that the strains clustered into 9 CCs (CC398, CC7, CC59, CC1, CC5, CC72, CC15, CC20, CC78) and 4 singletons (Figure 1). The largest cluster was CC398 with 14 isolates, followed by CC7 with 13 isolates, CC59 with 12 isolates, CC1 with 9 isolates, CC5 with 8 isolates, CC72 with 8 isolates, CC15 with 2 isolates, CC20 with 2 isolates and CC78 with 2 isolates.

SCC*mec* typing was performed on 15 MRSA isolates. Among them, only two types (type IV and V) were found. Two thirds of them were type IV (66.7%, 10/15), and one third were type V (33.3%, 5/15).

There was a strong association observed between specific ST and *spa* types. The ST398 genotype was associated mainly with *spa t*571 (6/14) and *spa t*034 (4/14), less frequently with three types: *t*2582, *t*6606, and *t*7160. The ST7 genotype was mainly linked with *t*091 (9/13), less frequently with *t*796, *t*1685, and *t*14204. The ST59 genotype was associated primarily with *spa t*172 (5/12) and *t*437 (4/12). All the ST188 genotype was associated with *spa t*189.

### Antimicrobial susceptibility testing

The antimicrobial resistance profiles of 71 *S. aureus* isolates according to MLST were listed in Table 2. All the strains were susceptible to vancomycin, linezolid, nitrofurantoin, rifampicin, tigecycline and quinupristin/dalfopristin. Resistance to penicillin was observed in the majority (84.5%), followed by erythromycin (35.2%), clindamycin (29.6%), tetracycline (22.5%), cefoxitin (21.1%), ampicillin (21.1%) and trimethoprim-sulfamethoxazole (16.9%). The resistance rates to other antibiotics tested were less than 6%, including 5.6% to gentamicin, 4.2% to levofloxacin, 4.2% to ciprofloxacin, and 2.8% to moxifloxacin.

**Table 2:** Antimicrobial susceptibility profiles among the molecular types of 71 *S. aureus* isolates recovered from breast milk.

**Molecular type**	**Isolates (*n*)**	**FOX (%)**	**LZD (%)**	**CIP (%)**	**DA (%)**	**E (%)**	**SXT (%)**	**MOF (%)**	**FD (%)**	**V (%)**	**TET (%)**	**P (%)**	**RD (%)**	**LEV (%)**	**AMP (%)**	**GM (%)**	**Q/D (%)**	**TGC (%)**
ST398	14	35.7	0	0	21.4	21.4	7.1	0	0	0	7.1	100	0	0	35.7	0	0	0
ST7	13	0	0	0	23.1	23.1	15.4	0	0	0	61.5	100	0	0	0	15.4	0	0
ST59	12	41.7	0	0	33.3	41.7	0	0	0	0	41.7	83.3	0	0	41.7	0	0	0
ST188	5	20	0	20	20	40	40	20	0	0	0	100	0	20	20	20	0	0
ST6	5	0	0	0	0	0	20	0	0	0	0	60	0	0	0	0	0	0
ST1	3	33.3	0	33.3	0	33.3	33.3	33.3	0	0	0	66.7	0	33.3	33.3	0	0	0
ST5	3	0	0	0	33.3	33.3	66.7	0	0	0	0	66.7	0	0	0	33.3	0	0
ST15	2	0	0	0	0	50	0	0	0	0	0	100	0	0	0	0	0	0
ST20	2	0	0	0	100	100	0	0	0	0	0	50	0	0	0	0	0	0
ST88	2	100	0	0	50	50	100	0	0	0	50	100	0	0	100	0	0	0
ST615	2	50	0	0	50	50	0	0	0	0	0	50	0	0	50	0	0	0
ST630	2	0	0	0	50	50	0	0	0	0	50	100	0	0	0	0	0	0
ST8	1	0	0	0	100	100	0	0	0	0	0	100	0	0	0	0	0	0
ST12	1	0	0	0	0	0	0	0	0	0	0	100	0	0	0	0	0	0
ST22	1	0	0	0	100	100	0	0	0	0	0	100	0	0	0	0	0	0
ST25	1	0	0	0	100	100	100	0	0	0	0	100	0	0	0	0	0	0
ST508	1	0	0	100	100	100	0	0	0	0	0	0	0	100	0	0	0	0
ST1290	1	0	0	0	0	0	0	0	0	0	0	0	0	0	0	0	0	0
Total	71	21.1	0	4.2	29.6	35.2	16.9	2.8	0	0	22.5	84.5	0	4.2	21.1	5.6	0	0

Among the 71 S. *aureus* isolates, 23 (32.4%) strains were resistant to ≥3 antibiotics, including 4 (5.6%) MRSA and 19 (26.8%) MSSA strains. In the MSSA strains, eight (11.2%) strains were resistant to 3 antibiotics and mostly resistant to penicillin, erythromycin and clindamycin(Supplementary Table 1), seven (9.9%) strains showed resistance to 4 antibiotics, and four (5.6%) strains were resistant to ≥5 antibiotics, however, only four MRSA strains were found to be resistant to at least three antibiotics.

### Virulence gene profiles

The distribution of 33 putative virulence genes varied among the 71 S. *aureus* strains according to STs (Table 3). All of these virulence genes except *lukM* and *etb* genes were identified within multiple isolates, and all isolates exhibited simultaneous carriage of at least 5 virulence genes. Thirty-nine (54.9%, 39/71) isolates harbored ≥10 tested virulence genes, among which were 2 isolate with 20 genes, 1 isolates with 19 genes, 1 isolates with 18 genes, 3 isolates with 17 genes, 2 isolates with 16 genes, 1 isolates with 15 genes, 7 isolates with 14 genes, 6 isolates with 13 genes, 1 isolates with 12 genes, 7 isolates with 11 genes and 8 isolates with 10 genes. Compared with MSSA isolates, the carriage rates for *arcA* and *seq* genes in MRSA isolates were significantly higher, while those of *sdrD* and *lukE* were significantly lower. The *pvl* gene was detected in 8 strains, which represented 5 different STs, with ST59 being the most common.

**Table 3:** Frequencies of virulence genes among the molecular types of 71 *S. aureus* isolates recovered from breast milk.

**Molecular type**	**Isolates (n)**	***pvl***	***hla***	***hlb***	***hlg***	***hlg2***	***icaA***	***clfA***	***sdrC***	***sdrD***	***sdrE***	***bsa***	***lukE***	***lukM***	***tsst***	***eta***	***etb***	***arcA***
ST398	14	0	100	100	100	42.9	100	100	100	0	78.6	0	0	0	0	0	0	14.3
ST7	13	0	100	15.4	38.5	100	100	100	100	100	7.7	7.7	13	0	7.7	15.4	0	15.4
ST59	12	33.3	100	100	0	100	100	100	100	0	91.7	0	0	0	0	0	0	33.3
ST188	5	20	100	60	40	100	100	100	100	0	100	20	100	0	0	0	0	20
ST6	5	0	100	40	20	100	100	100	100	100	80	100	100	0	20	40	0	0
ST1	3	33.3	100	33.3	33.3	100	100	100	100	66.7	33.3	100	100	0	66.7	33.3	0	0
ST5	3	0	100	33.3	0	100	100	100	0	100	100	0	100	0	33.3	0	0	0
ST15	2	0	100	0	0	100	100	100	100	100	50	0	100	0	0	0	0	0
ST20	2	0	100	50	0	100	100	100	100	50	100	0	100	0	50	0	0	0
ST88	2	0	100	100	0	100	100	100	100	100	0	0	100	0	0	0	0	50
ST615	2	1	100	100	100	100	100	100	100	100	100	0	100	0	100	0	0	0
ST630	2	0	100	100	0	100	100	100	100	50	50	0	0	0	0	0	0	0
ST8	1	0	100	100	0	100	100	100	100	100	100	100	100	0	0	0	0	0
ST12	1	0	100	100	100	100	100	100	100	100	100	0	100	0	0	0	0	0
ST22	1	100	100	100	100	100	100	100	100	100	100	0	0	0	0	0	0	0
ST25	1	0	100	0	0	100	100	100	100	0	100	100	100	0	0	0	0	0
ST508	1	0	100	0	100	100	100	100	100	0	100	0	0	0	0	0	0	0
ST1290	1	0	100	0	0	100	100	100	0	100	100	0	100	0	0	0	0	0
Total	71	11.3	100	63.4	39.4	88.7	100	100	94.4	49.3	67.6	16.9	57.8	0	11.3	7	0	14.1

Adhesion genes were present in most of the *S. aureus* isolates; 100% carried the *icaA* and *clfA* genes, 94.4% harbored *sdrC*, 67.6% carried *sdrE* and 49.3% carried *sdrD*.

The most prevalent toxin genes detected were *hla* (100%), *hlg*2 (88.7%), *lukE* (57.8%), *hlb* (43.7%). The carriage rates for *tsst* (11.3%) and *eta* (7.0%) in breast milk isolates were low.

The carriage of staphylococcal enterotoxin genes was a strong association with MLST profiles.

Thirteen classical enterotoxin genes (*sea, seb, sec, sed, see, seg, seh, sei, sem, sen, seo, seq, sek*) were detected within these strains (Table 4). Overall, each enterotoxin gene was found in multiple *S. aureus* isolates, ranging from 5.6 to 31.0%. No enterotoxin gene was found in ST1290 and ST630 isolates. The *see*-*sep* genes were present in the ST7 strains, whereas, the *sed*-*sej* genes were present in ST5 and ST615 strains. All ST5, ST20, ST22, ST25, ST26, ST508, and ST615 strains harbored *seg*-*sei*-*sem*-*sen*-*seo* genes, but ST59 isolates mainly carried *seb*-*sek*-*seq* genes.

**Table 4:** Frequencies of staphylococcal enterotoxin genes among the molecular types of 71 *S. aureus* isolates recovered from breast milk.

**Molecular type**	**Isolates (*n*)**	***sea***	***seb***	***sec***	***sed***	***see***	***seg***	***seh***	***sei***	***sej***	***sek***	***seq***	***sel***	***sem***	***sen***	***seo***	***sep***
ST398	14	0	0	14.3	14.3	0	7.1	0	0	0	0	0	0	21.4	7.1	7.1	14.3
ST7	13	23.1	0	15.4	15.4	53.9	15.4	0	0	7.7	0	0	0	30.8	15.4	23.1	46.2
ST59	12	33.3	75	0	0	0	25	0	8.3	0	66.7	66.7	16.7	8.3	8.3	8.3	0
ST188	5	40	40	0	20	0	40	20	20	0	0	0	40	40	20	20	0
ST6	5	60	0	0	0	0	0	0	0	20	20	0	20	20	20	20	0
ST1	3	33.3	33.3	66.7	0	0	0	100	0	33.3	100	66.7	33.3	33.3	33.3	66.7	0
ST5	3	0	0	33.3	100	0	100	0	100	100	33.3	33.3	33.3	100	100	100	0
ST15	2	0	0	0	0	0	50	0	0	0	0	0	0	0	0	0	0
ST20	2	0	0	0	0	0	100	0	100	0	0	0	50	100	100	100	0
ST88	2	0	0	0	0	100	0	0	0	0	0	0	0	0	0	0	0
ST615	2	0	50	50	100	0	100	0	100	100	100	50	50	50	100	100	0
ST630	2	0	0	0	0	0	0	0	0	0	0	0	0	0	0	0	0
ST8	1	0	0	0	100	0	0	0	0	100	0	0	0	100	0	0	0
ST12	1	0	0	100	0	100	0	0	0	0	0	0	100	0	0	0	0
ST22	1	0	100	0	0	0	100	0	100	0	100	0	100	100	100	100	0
ST25	1	0	100	0	0	0	100	0	100	0	0	0	0	100	100	100	0
ST508	1	0	0	0	0	0	100	0	0	0	0	0	0	100	100	100	0
ST1290	1	0	0	0	0	0	0	0	0	0	0	0	0	0	0	0	0
Total	71	18.3	21.1	12.7	15.5	14.1	26.8	5.6	15.5	12.7	22.5	16.9	15.5	31	23.9	26.8	11.3

### Molecular characteristics of the prevalent clone ST398

In this study, ST398 (19.7%, 14/71) was found to be the most prevalent clone, including 5 MRSA and 9 MSSA isolates, which was associated primarily with *spa t*571 (6/14) and *spa t*034 (4/14), less frequently with three types: *t*2582, *t*6606, and *t*7160. Among 14 ST398 stains, all were susceptible to vancomycin, linezolid, nitrofurantoin, ciprofloxacin, moxifloxacin, levofloxacin, rifampicin, gentamicin, tigecycline and quinupristin/dalfopristin. The highest levels of resistance were observed for penicillin (100%), cefoxitin (35.7%), and ampicillin (35.7%). The resistance rates to other antibiotics tested were 21.4% to clindamycin,21.4% to erythromycin,7.1% to trimethoprim-sulfamethoxazole and 7.1% to tetracycline. In addition, there were no significant differences in antibiotic sensitivities between ST398 and non-ST398 isolates (Supplementary Table 2).

All ST398 isolates exhibited *icaA, clfA, sdrC, hla, hlb*, and *hlg* genes, however, the frequency of carriage for *hlg2, sdrD, lukE, seb*, and *sek* was significantly lower than that for non-ST398 isolates (Supplementary Table 3). In addition, there were no significant differences on the positive rate of *pvl* between ST398 and non-ST398 strains.

## Discussion

Breast milk is considered to be the best source of nutrients for infant growth and development in the world. However, breast milk isn't always sterile and may contain pathogenic bacteria that could cause infections especially in premature infants. *S. aureus* is a common colonizer of skin and mucous membranes in human and infection by *S. aureus* is often occur following breaks in skin or mucosal barriers. *S. aureus* is one of the most frequently isolated pathogenic bacteria in breast milk (Barbosa-Cesnik et al., 2003) and could cause a wide variety of infections including pneumonia, sepsis, skin lesion and food poisoning among infants. Given these dangerous consequences, it is urgent to understand the prevalence, molecular characteristics and virulence profiles of *S. aureus* isolates from breast milk in order to implement right measures to control infection and transmission.

The detection rate of *S. aureus* in breast milk varies substantially worldwide, ranging from 2.5 to 100% in different countries. In Brazil, studies on the frequency of *S. aureus* in breast milk have shown differences between 2.5 and 34%. In the present study, 71 (6.4%, 71/1102) *S. aureus* strains were isolated from 1102 breast milk samples and 15 (1.4%, 15/1102) have identified as MRSA. This indicates that *S. aureus* is an important pathogenic bacterium in breast milk now and suggests the urgent need for active surveillance of *S. aureus* and MRSA infection and transmission in mothers and infants.

In the current study, ST398 (19.7%, 14/71), ST7 (18.3%, 13/71), and ST59 (16.9%, 12/71) were the three predominant STs, accounting for 54.9% of all *S. aureus* isolates. Surprisingly, ST398 was found to be the most frequently represented ST in breast milk. ST398 is a typical livestock-associated type (Graveland et al., 2011; Qiao et al., 2014), which first observed among pigs and pig farmers in Netherlands in 2003, then found in Austria, Germany and Denmark (Fluit, 2012). Afterward, it became the overwhelmingly dominant lineage in Europe and North America. Previous studies showed that patients carrying this type were usually in contact with animal reservoirs of these MRSA. Recently, ST398 clones were found in different samples of patients in China, including sputum, blood, pus and secretion (Zhao et al., 2012; He et al., 2013; Song et al., 2017). Moreover, breast milk also became the source of ST398 in our study and favored the transmission between mothers and infants. There was no evidence shown that all the mothers had ever been exposed to livestock because of the lack of adequate information. It is very difficult to speculate on the origins of these isolates because of the absence of epidemiological data linking these to animals. However, livestock-associated *S. aureus* usually harbored an intact beta-toxin gene (*hlb*) and no lysogenic prophages encoding the immune evasion complex genes (*sea, sep, sak, scn*, and *chp* genes) (van Wamel et al., 2006). Among ST398 isolates in this study, they all harbored an intact *hlb* gene and didn't carry *sea* and *sep* genes. This was powerful evidence that these strains were of animal origin. Two strains lacked all the immune evasion complex genes, and others harbored one, two or three of *sak, scn* and *chp* genes. The *sak, scn* and *chp* genes are usually encoded by *hlb*-disrupting bacteriophages, this suggested the other isolates may harbor prophages integrated somewhere else besides the *hlb* gene. ST7, found in a total of 13 MSSA isolates, was the second common ST in the present study. ST7 has also been reported to be one of the most dominant MSSA genotypes in invasive CA-SA infection in Chinese children (Qiao et al., 2014). Another study in our group showed ST7 also was one of the common genotypes causing bovine mastitis in Shanghai between 2014 and 2015 (Li et al., 2017). ST7, which was considered as a pandemic clone, have arisen in communities and spread across the country. In addition, it is well known ST59 is the most predominant CA-MRSA clone in the Asia-Pacific region, including Taiwan and Hong Kong (Chuang and Huang, 2013). In China, previous studies revealed that ST59-MRSA-IV was the major lineage accounting for up to two-thirds of isolates (Geng et al., 2010). CC59 was also reported to be the most common clonal complex among the patients with SSTIs (Yu et al., 2015). Similar to these findings, ST59 were also found to be one of the dominant types in our study and still spread widely in the communities. From these results, our study provided evidence for the existence of two different lineages of *S. aureus* in breast milk in China: LA-SA and CA-SA.

The invasive potential of *S. aureus* largely depends on the carriage of a battery of virulence factors associated with adhesion, acquisition of nutrients, tissue penetration, evasion of host defenses and toxin-mediated responses (Dinges et al., 2000; Bubeck Wardenburg et al., 2007; Diep and Otto, 2008). Consistent with other findings, the prevalence of *icaA, clfA, sdrC* among *S. aureus* isolated from breast milk in our study were high (100, 100, and 94.4%), supporting the statement that adherence of *S. aureus* to host cells was the crucial initial step for bacterial pathogenicity. The distribution of some virulence genes, especially enterotoxin genes, was correlated with the different *S. aureus* lineages. All ST398 isolates harbored *hlg* gene, but the frequency of carriage for *hlg2, sdrD, lukE, seb*, and *sek* was significantly lower than that for non-ST398 isolates (*P* < 0.05). Compared with other ST isolates, ST7 isolates harbored less *hlb* but more *see*-*sep*. None of ST59 isolates carried *hlg, sdrD*, and *lukE*, but contained *seb*-*sek*-*seq* genes, which was significantly higher than that among non-ST59 isolates. In addition, All ST5, ST20, ST22, ST25, ST26, ST508, and ST615 strains harbored *seg*-*sei*-*sem*-*sen*-*seo* genes. These findings implied distinctive virulence genes have different roles in the pathogenicity of *S. aureus* lineages.

In conclusion, our findings showed that breast milk was a reservoir for LA-SA (ST398) and CA-SA(ST59) and was likely a vehicle for transmission of multidrug-resistant *S. aureus* and MRSA lineages. This is a serious public health risk and highlights the need to implement good hygiene practices. Additional studies are required to assess the source of contamination of breast milk samples and the risk of infection to babies.

## Author contributions

XW: designed the studies and obtained funding; XW, XL, YZ, XZ, and WH: performed the experiments and/or analyzed the data; XW and XL: wrote the manuscript.

### Conflict of interest statement

The authors declare that the research was conducted in the absence of any commercial or financial relationships that could be construed as a potential conflict of interest.
