# Supplementary material for: Breast Milk Is a Potential Reservoir for Livestock-Associated Staphylococcus aureus and Community-Associated Staphylococcus aureus in Shanghai, China
Source: Front Microbiol. 2018 Jan 11;8:2639. doi: 10.3389/fmicb.2017.02639 (PMC5768657; doi:10.3389/fmicb.2017.02639)
Supplement: Supplementary file 2 [file DataSheet2.docx]

**Supplementary Table 2.** Antimicrobial susceptibility profiles of *S.aureus* isolates, ST398 and non-ST398 isolates.

|  | *S.aureus*(n=71),R^a^ (%) | ST398 *(*n=14*)*, R^a^ (%) | Non-ST398(n=57),R^a^ (%) | P value^b^ |
| --- | --- | --- | --- | --- |
| FOX | 21.1 | 35.7 | 17.5 | >0.05 |
| LZD | 0 | 0 | 0 |  |
| CIP | 4.2 | 0 | 5.3 | >0.05 |
| DA | 29.6 | 21.4 | 31.6 | >0.05 |
| E | 35.2 | 21.4 | 38.6 | >0.05 |
| SXT | 16.9 | 7.1 | 19.3 | >0.05 |
| MOF | 2.8 | 0 | 3.5 | >0.05 |
| FD | 0 | 0 | 0 |  |
| V | 0 | 0 | 0 |  |
| TET | 22.5 | 7.1 | 26.3 | >0.05 |
| P | 84.5 | 100 | 80.7 | >0.05 |
| RD | 0 | 0 | 0 | >0.05 |
| LEV | 4.2 | 0 | 5.3 | >0.05 |
| AMP | 21.1 | 35.7 | 17.5 | >0.05 |
| GM | 5.6 | 0 | 7 | >0.05 |
| Q/D | 0 | 0 | 0 |  |
| TGC | 0 | 0 | 0 |  |

^a^R = Resistance.

^b^The resistance rates of antimicrobials among ST398 were compared with those among non-ST398 isolates.

**Supplementary Table 3.** Frequencies of virulence genes among *S.aureus* isolates, ST398 and non-ST398 isolates.

| Virulence gene | *S.aureus*(n=71) | ST398 *(*n=14*)* | Non-ST398(n=57) | P value^a^ |
| --- | --- | --- | --- | --- |
| *pvl* | 11.3 | 0 | 14.0 | >0.05 |
| *hla* | 100 | 100 | 100 |  |
| *hlb* | 63.4 | 100 | 54.4 | <0.05 |
| *hlg* | 39.4 | 100 | 24.6 | <0.05 |
| *hlg2* | 88.7 | 42.9 | 100 | <0.05 |
| *icaA* | 100 | 100 | 100 |  |
| *clfA* | 100 | 100 | 100 |  |
| *sdrC* | 94.4 | 100 | 93.0 | >0.05 |
| *sdrD* | 49.3 | 0 | 61.4 | <0.05 |
| *sdrE* | 67.6 | 78.6 | 64.9 | >0.05 |
| *bsa* | 16.9 | 0 | 21.1 | >0.05 |
| *lukE* | 57.7 | 0 | 71.9 | <0.05 |
| *lukM* | 0 | 0 | 0 |  |
| *tsst* | 11.3 | 0 | 14 | >0.05 |
| *eta* | 7 | 0 | 8.8 | >0.05 |
| *etb* | 0 | 0 | 0 | >0.05 |
| *arcA* | 14.1 | 14.3 | 14 | >0.05 |
| *sea* | 18.3 | 0 | 22.8 | >0.05 |
| *seb* | 21.1 | 0 | 26.3 | <0.05 |
| *sec* | 12.7 | 14.3 | 12.3 | >0.05 |
| *sed* | 15.5 | 14.3 | 15.8 | >0.05 |
| *see* | 14.1 | 0 | 17.5 | >0.05 |
| *seg* | 26.8 | 7.1 | 31.6 | >0.05 |
| *seh* | 5.63 | 0 | 7.0 | >0.05 |
| *sei* | 15.5 | 0 | 19.3 | >0.05 |
| *sej* | 12.7 | 0 | 15.8 | >0.05 |
| *sek* | 22.5 | 0 | 28.1 | <0.05 |
| *seq* | 16.9 | 0 | 21.1 | >0.05 |
| *sel* | 15.5 | 0 | 19.3 | >0.05 |
| *sem* | 31 | 21.4 | 33.3 | >0.05 |
| *sen* | 23.9 | 7.1 | 28.1 | >0.05 |
| *seo* | 26.8 | 7.1 | 31.6 | >0.05 |
| *sep* | 11.3 | 14.3 | 10.5 | >0.05 |

^a^ The positive rates of virulence genes among ST398 were compared with those among non-ST398 isolates.
